# *Rhipicephalus bursa* Sialotranscriptomic Response to Blood Feeding and *Babesia ovis* Infection: Identification of Candidate Protective Antigens

**DOI:** 10.3389/fcimb.2018.00116

**Published:** 2018-05-04

**Authors:** Sandra Antunes, Joana Couto, Joana Ferrolho, Fábio Rodrigues, João Nobre, Ana S. Santos, M. Margarida Santos-Silva, José de la Fuente, Ana Domingos

**Affiliations:** ^1^Global Health and Tropical Medicine, Instituto de Higiene e Medicina Tropical, Universidade Nova de Lisboa, Lisbon, Portugal; ^2^Instituto de Higiene e Medicina Tropical, Universidade Nova de Lisboa, Lisbon, Portugal; ^3^Instituto Nacional de Investigação Agrária e Veterinária, Pólo de Santarém, Vale de Santarém, Portugal; ^4^Instituto Nacional de Saúde Doutor Ricardo Jorge, Centro de Estudos de Vectores e Doenças Infecciosas Dr. Francisco Cambournac (CEVDI/INSA), Águas de Moura, Portugal; ^5^SaBio, Instituto de Investigación en Recursos Cinegéticos IREC-CSIC-UCLM-JCCM, Ciudad Real, Spain; ^6^Department of Veterinary Pathobiology, Center for Veterinary Health Sciences, Oklahoma State University, Stillwater, OK, United States

**Keywords:** sialotranscriptomics, *Rhipicephalus bursa*, *Babesia* spp., RNA interference, vaccine, vector-pathogen interactions

## Abstract

Ticks are among the most prevalent blood-feeding arthropods, and they act as vectors and reservoirs for numerous pathogens. Sialotranscriptomic characterizations of tick responses to blood feeding and pathogen infections can offer new insights into the molecular interplay occurring at the tick-host-pathogen interface. In the present study, we aimed to identify and characterize *Rhipicephalus bursa* salivary gland (SG) genes that were differentially expressed in response to blood feeding and *Babesia ovis* infection. Our experimental approach consisted of RNA sequencing of SG from three different tick samples, fed-infected, fed-uninfected, and unfed-uninfected, for characterization and inter-comparison. Overall, 7,272 expressed sequence tags (ESTs) were constructed from unfed-uninfected, 13,819 ESTs from fed-uninfected, and 15,292 ESTs from fed-infected ticks. Two catalogs of transcripts that were differentially expressed in response to blood feeding and *B. ovis* infection were produced. Four genes coding for a putative vitellogenin-3, lachesin, a glycine rich protein, and a secreted cement protein were selected for RNA interference functional studies. A reduction of 92, 65, and 51% was observed in *vitellogenin-3, secreted cement*, and *lachesin* mRNA levels in SG, respectively. The *vitellogenin-3* knockdown led to increased tick mortality, with 77% of ticks dying post-infestation. The reduction of the secreted cement protein-mRNA levels resulted in 46% of ticks being incapable of correctly attaching to the host and significantly lower female weights post-feeding in comparison to the control group. The *lachesin* knockdown resulted in a 70% reduction of the levels associated with *B. ovis* infection in *R. bursa* SG and 70% mortality. These results improved our understanding of the role of tick SG genes in *Babesia* infection/proliferation and tick feeding. Moreover, lachesin, vitellogenin-3, and secreted cement proteins were validated as candidate protective antigens for the development of novel tick and tick-borne disease control measures.

## Introduction

Ticks are widely distributed obligate hematophagous ectoparasites, which have recognized effects on host species. During blood feeding, ticks secrete varying substances into the host bloodstream acting as remarkable vectors of numerous pathogens, some of which can cause severe diseases in vertebrate hosts, including humans (Jongejan and Uilenberg, 2004; Domingos et al., 2013; Sonenshine and Michael Roe, 2014). Reflecting the progress of feeding, salivary glands (SG) increase ~25-fold in mass and protein content, as the glands are responsible for the production of complex saliva that is capable of quelling host innate and adaptive immune responses (Sauer et al., 2000; Kazimírová and Stibraniova, 2013; Kotál et al., 2015; Šimo et al., 2017). SG play an essential role in tick survival and success as parasites by modulating host haemostasis and complement systems (Sauer et al., 2000; Francischetti et al., 2009; Kazimírová and Stibraniova, 2013). In addition to being involved with osmoregulation (Kaufman, 2010), this tissue is also responsible for the production of cement, which is an adhesive substance that surrounds the mouthparts and the host skin that ensures tick attachment (Sauer et al., 2000; Francischetti et al., 2009; Kazimírová and Stibraniova, 2013; Šimo et al., 2017). SG are also pivotal in tick pathogen interactions, because pathogens need to cross the physical barrier of SG epithelium and endure the salivary biochemical environment to gain access to the next host. Remarkably, to increase their proliferation and transmission, pathogens adapted to SG in a way that exploits tick salivary molecules (Ramamoorthi et al., 2005; Kaufman, 2010). Therefore, these features make SG an exceptional target for the identification of new candidate protective antigens that are relevant to biological functions associated with tick development, fertility, feeding, and pathogen infection and transmission (Merino et al., 2013; Shahein et al., 2013).

Research that examined tick SG made the characterization of a large number of tick salivary compounds possible, but the function of several of these molecules remains unknown (Francischetti et al., 2009). The sialomes of some tick species have been described (Francischetti et al., 2008, 2011; Anatriello et al., 2010; Karim et al., 2011; Tan et al., 2015; de Castro et al., 2016; Moreira et al., 2017), and this information represents an important data source for functional studies and analyses of gene expression dynamics during tick feeding. Moreover, high-throughput technologies have also enabled researchers to study the effects of sex, physiological stages, and different tick statuses such as the presence of pathogens in tick tissues (Chmelar et al., 2016).

*Rhipicephalus bursa* is a multi-host tick that is mainly associated with ruminants, but it can occasionally parasitize other animals such as wild ungulates and small mammals (Walker et al., 2000; de la Fuente et al., 2004; Santos-Silva et al., 2011; Mihalca et al., 2012). *R. bursa* is recognized as the primary vector of *Babesia ovis* (Moltmann et al., 1982a), but it transmits other pathogens such as *Rickettsia* spp. and *Anaplasma* spp. (Raele et al., 2015; Dahmani et al., 2016; Ferrolho et al., 2016b), thus demonstrating its importance in animal health, particularly in livestock. *B. ovis*, an intraerythrocytic apicomplexan parasite, is the main etiological agent of ovine babesiosis, which is a tick-borne disease of small ruminants, and its geographical distribution overlaps with that of *R. bursa* (Walker et al., 2000; Ranjbar-Bahadori et al., 2012; Erster et al., 2015; Ferrolho et al., 2016a). This highly pathogenic organism is characterized by low parasitaemia, and it causes severe infections (Habela et al., 1990; Sevinc et al., 2013; Hurtado et al., 2015). *B. ovis* is extremely well adapted to the vector, and it survives in the tick during several successive generations (Yeruham et al., 2001) using horizontal and vertical transmission (Friedhoff, 1988). Microscopy studies in the 1980's discovered that the *B. ovis* cycle within the tick is similar to other *Babesia* spp. (Moltmann et al., 1982a,b). Briefly, *Babesia* penetrates the tick midgut, undergoes meiosis, and differentiates into motile ookinetes that propagate via haemolymph to reach all tick organs. *B. ovis* kinetes reach SG within 48 h post-infestation, and they undergo a final step of multiplication to produce sporozoites (Moltmann et al., 1982a; Antunes et al., 2017). Adult ticks are the main vector, and both females and males are implicated in the transmission of the hemoparasite. However, females present a higher threat due to transovarial transmission and extended feeding periods (Friedhoff, 1988).

The importance of the *R. bursa*-*B. ovis* system was emphasized in a disease outbreak that resulted in animal morbidity and mortality (Hurtado et al., 2015). Pathogen and vector control methods are limited to the common usage of imidocarb dipropionate (to manage animal disease) and acaricides (McHardy et al., 1986; Belloli et al., 2006; Domingos et al., 2013). Safer and effective alternatives are urgently needed, including the development of vaccines that may reduce tick infestations and block pathogen transmission (Merino et al., 2013; Liu and Bonnet, 2014; Neelakanta and Sultana, 2015). Studies of the molecular interactions associated with the tick-pathogen interface represent a bridge for the identification of antigenic targets to implement vaccination strategy. Information about the *R. bursa* and *B. ovis* interactome is scarce. Thus, in the present study, SG of *R. bursa* adult females were used to assess the transcriptomic response to blood feeding and *B. ovis* infection. Fed-infected, fed-uninfected, and unfed-uninfected female ticks were produced, SG were isolated and used for RNA extraction. RNA-seq and *de novo* transcriptome assembly approaches were used to construct the sialotranscriptome of fed-infected, fed-uninfected, and unfed-uninfected *R. bursa* specimens. These catalogs were analyzed, and four genes were selected for further functional studies, thus allowing the evaluation of encoded proteins for inclusion in anti-tick and tick-borne pathogen vaccines. These data are essential for vaccinomics pipelines, which could enhance our knowledge of the dynamic processes that occur at the tick-pathogen-host interface.

## Materials and methods

### Ethics statement

Animal experiments were conducted with the approval of the Divisão Geral de Alimentação e Veterinária (DGAV), Portugal, under Art° 49, Portaria n°1005/92 from 23rd October (permit number 0421/2013) and the Council of Ethics of the Instituto de Higiene e Medicina Tropical (IHMT). Animals were maintained and manipulated following protocols compliant with the national and European Animal Welfare legislation, in frame with DL 113/2013 and Directive 2010/63/EUbased on the principle of the Three R's, to replace, reduce, and refine the use of animals for scientific purposes.

### *Rhipicephalus bursa* colony

*R. bursa* colony was established under laboratory conditions and further maintained. For colony initiation, adult ticks were collected either in naturally infested domestic animals or by dragging/flagging the vegetation and kept in a chamber regulated at 25 ± 1°C, 70 ± 10% relative humidity and a photoperiod of 16:8 (light: dark). During oviposition, the dark period was increased to improve female egg laying. After oviposition, each female and a sample of eggs were tested by conventional PCR for pathogens detection (*Babesia* spp.*, Anaplasma* spp., *Ehrlichia* spp.) during two generations using the protocols and primers described elsewhere (Inokuma et al., 2000; de la Fuente et al., 2003; Aktaş et al., 2005; Harrus et al., 2011). Ticks were fed on Hyla breed rabbits at Centro de Estudos de Vetores e Doenças Infeciosas, Instituto Nacional de Saúde Doutor Ricardo Jorge (CEVDI/INSA) in appropriate conditions. Ten lineages of *R. bursa* were selected in order to reduce interbreeding.

### *In Vitro Babesia ovis* cultures

*In vitro B. ovis* cultures were established at IHMT in biosafety level 2 facilities, following a protocol adapted from Vega et al. (1985). Briefly, cryopreserved *B. ovis* (Israeli strain) infected red blood cells (RBC) were used to initiate the culture. *B. ovis* merozoites were cultured in lamb erythrocytes maintained in 20% lamb serum-containing medium, in an atmosphere of 5% CO_2_/2% O_2_/93% N_2_ at 37°C, as described elsewhere (Horta et al., 2014). Half of the medium was replaced daily and cultures monitored for parasitaemia by preparing thin blood smears stained with Hemacolor® Rapid staining of blood smear (EMD Millipore, Darmstadt, Germany). Intraerythrocytic parasites were observed under a 400x original magnification of a Nikon eclipse 80i fluorescence microscope.

### Salivary glands and RNA samples for RNA-Seq

#### Fed and unfed *R. bursa*

Thirty adult female ticks were carefully removed from the rabbits ear 10–12 days post attachment. Equally, thirty unfed adult female ticks were also obtained. Ticks were individually rinsed in distilled water, after in 75% (v/v) ethanol, once more in water and dissected under a stereoscopic microscope at 4x magnification (Motic SMZ-171B, China) using sterile conditions in ice-cold phosphate-buffered saline (PBS). The SG were stored in RNAlater (Ambion, Austin, TX, USA) and afterwards pooled, resulting in two samples for the fed condition and other two for the unfed. Total RNA was extracted from each sample using Tri-reagent (Sigma–Aldrich, St. Louis, MO, USA). RNA quantity was estimated using the ND-1000 Spectrophotometer (NanoDrop ND1000, Thermo Fisher Scientific, Whaltman, MA, USA).

#### Fed-*B. ovis* infected *R. bursa*

A batch of 60 female ticks were inoculated with *B. ovis* in the trochanter—coxae articulation and allowed to feed on rabbits. After drop off, SG were carefully isolated and DNA/RNA extracted has mentioned previously. Genomic DNA was used to amplify a 549 bp fragment of *B. ovis 18S* ribosomal DNA (18S rRNA) using primers and conditions described elsewhere (Aktaş et al., 2005). RNA from positive samples (Supplementary Figure 1) were used for the production of two RNA pools with fifteen samples each. All samples were promptly shipped in dry ice to Parque Cientifico de Madrid for sequencing. The tick infection model and vector competence was evaluated. *B. ovis* inoculated *R. bursa* were allowed to feed in a naïve lamb. The lamb was monitored every two days for babesiosis clinical symptoms and blood collected for *B. ovis* detection by PCR (Supplementary Figure 1) using the above mentioned conditions. After 8 days, the ticks were recovered for analysis.

### RNA-Seq

RNA quality was assessed using an Agilent RNA 6000 bioanalyzer (Agilent Technologies, CA, USA). Libraries preparation was performed with “NEBNext Ultra Directional RNA Library Prep” kit (New England Biolabs, Ipswich, MA, USA) following manufacturer instructions. Briefly, prior to cDNA library construction magnetic beads with oligo (dT) were used to enrich poly (A) mRNA from 1 μg of total-RNA. Next, the purified mRNAs were disrupted into short fragments, and double-stranded cDNAs were immediately synthesized. The cDNAs were subjected to end-repair and adenilation, then connected with sequencing adapters. Suitable fragments, purified by size selection protocol with AMPure XP beads (Beckman Coulter), were selected as templates for PCR amplification. The final library sizes and qualities were evaluated electrophoretically using an Agilent High Sensitivity DNA kit (Agilent Technologies, CA, USA); the mean fragment size was 510 bp. Subsequently, the library was sequenced using a HiSeq 2500 sequencer (Illumina, CA, USA) in rapid run mode. Cluster generation was performed, followed by 2 × 100 cycle sequencing reads separated by a paired-end turnaround. Image analysis was performed using the HiSeq control software version 1.8.4. The raw fastq files were deposited in the Sequence Read Archives (SRA) of the National center for Biotechnology information (NCBI) under the accession numbers SRR4428986, SRR4428987 and SRR4428988, Biosamples SAMN05916213, SAMN05916214, and SAMN05916215, regarding the unfed-uninfected, fed-uninfected and fed-infected populations, respectively, of Bioproject PRJNA348674. The Transcriptome Shotgun Assembly (TSA) projects have been deposited at DDBJ/EMBL/GenBank under the accessions GFZD00000000, GFZJ00000000, and GFZK00000000. The versions described in this paper are the first versions, GFZD01000000, GFZJ01000000, and GFZK01000000.

### Transcriptomic data of female *R. bursa* sialome

#### Assembly and analysis of transcripts

This project comprised *de novo* assembly of six transcriptomes. Three conditions and two replicas per condition: F, SG from fed ticks; NFni, SG from unfed-uninfected ticks; and Fi, SG from fed-*B. ovis* infected ticks. Subsequently, two comparisons were performed: F *vs*. NFni (response to blood feeding) and F *vs*. Fi (response to *B. ovis* infection). Quality analysis of the raw reads was done with Prinseq tool (Schmieder and Edwards, 2011). Pre-processing of reads included: (a) right trimming where quality < Q30; (b) left trimming of the first base; (c) filtering out reads with Ns; (d) quality analysis of the processed data. For each of the four transcriptomes three *de novo* assemblies were made with three different *k*-values using the *de novo* transcriptome assembler Oases (Velvet, version: 1.2.10) (Schulz et al., 2012). The annotation of each transcript was done based on the Basic Local Alignment Search Tool (BLAST) results comparing the transcript to a database of reference proteins. The set of reference proteins was selected from UniProt database from all the organisms belonging to the taxon “Ixodidae”. In total 76, 475 proteins were used as reference proteins. A set of unigenes for each sample was obtained. The assignment of each transcript to a protein was based on BLAST similarity. Rich functional annotation for each unigene extracted from the UniProt protein in which the read clustering process has been centered for this unigene is provided. Afterwards a unigene expression quantification was performed using eXpress. To compare the transcripts from the samples, the transcripts were clustered by protein. The protein driven transcript clusters that were done using UniProt proteins, were furtherly clustered by UniRef90 proteins. The mapping from the UniProt proteins to UniRef90 was done using UniProt retrieval tool. The quantification per UniRef90 cluster was calculated adding the quantification per protein included in each UniRef90 cluster. *P*-value calculation of the *Z*-test was based on the raw counts (total exon reads per gene). Genes were considered significantly differentially expressed if the *P*-value was below 0.05. Functional annotation of these genes was manually done by compiling information from UniProt, RefSeq, GO, Panther, KEGG, Pfam, and NCBI databases.

#### Gene ontology assignments

Functional data for each identified protein was obtained using Blast2GO platform version 4.0.7 available at https://www.blast2go.com (Conesa et al., 2005; Götz et al., 2008). Homology to the protein sequences was searched by BLAST against Arthropoda (nr subset) [arthropoda, taxa:6656] from 30.01.2017 as well as against to InterPro protein signature databases, using InterProScan. To retrieve gene ontology (GO) terms, a mapping step was performed gathering GO annotations and evidence codes (EC). Annotation to assign functional terms was performed next. At this step, the most specific and reliable annotation was considered. Finally, to map a set of annotations to high level GO terms, GO slim option was used. GO frequency charts were constructed using the Microsoft Office 2016 Excel tool. The most up and down-regulated genes in response to feeding and infection (*P* < 0.1) were analyzed using the same approach.

### Validation of RNA-Seq data

A total of 18 transcripts with differential regulation and belonging to different functional classes with a potential interference in response to blood feeding and *B. ovis* infection, were chosen for RNA-Seq validation through qPCR using the minimum information for publication of qPCR experiments (Bustin et al., 2009). Ten individual *R. bursa* SG, from each condition studied, were used to extract total RNA using the GRS FullSample Purification kit, Grisp™ (Porto, Portugal), which included DNAse treatment and 60 ng/μL of each sample were used to synthesize cDNA using the iScript™ cDNA Synthesis Kit (Bio-Rad, CA, USA). qPCR reactions of 10 μL were performed in triplicate using IQ™ SYBR® Green Supermix kit (Bio-Rad, CA, USA) in a CFX Connect™ Real-Time PCR Detection System (Bio-Rad). The cycling conditions were as follows: an initial cycle of denaturation at 95°C for 10 min; followed by 45 cycles of 95°C for 15s and temperature of each primer set for 45s. Fluorescence readings were taken at 62°C after each cycle and a dissociation curve (60–95°C) was performed. Negative controls were prepared with water. To determine the reaction efficiency standard curves were constructed with five-fold serial dilutions of cDNA from *R. bursa*. Reactions specificity was assured by the absence of PCR product in control reactions and by the dissociation curves (60–95°C) run at the end the cycling protocol. The average expression stability (*M*-value) of the reference genes, β *tubulin*, β *actin, elongation factor*, and *16S*, was assessed based in geNorm algorithm (Vandesompele et al., 2002) included in the CFX Manager™ Software (Bio-Rad, CA, USA) and gene relative quantification was evaluated using the CFX Manager™ Software including the *Pfaff* method (Pfaffl, 2001) using the above-mentioned reference genes for normalization. Normalized Cq-values were compared between conditions by Student's t test (*P* < 0.05). Primers were design using Primer3 platform (http://bioinfo.ut.ee/primer3-0.4.0/) and their conditions are summarized in Supplementary Table 1. Pearson's correlation was used to compare the expression values between RNA-Seq and qPCR methods for the 18 selected genes.

### RNA interference assays

#### Lamb infection with *B. ovis*

A six-month old lamb bred and maintained at the Instituto Nacional de Investigação Agrária e Veterinária (INIAV) animal facility was splenectomized and, 45 days after, intravenously inoculated with 1 mL of cryopreserved *B. ovis* culture with 9% parasitemia. The *B. ovis* infection was monitored daily by blood screening. Genomic DNA was extracted from lamb blood using the NZY Blood gDNA Isolation Kit (NZYTech, Lisboa, Portugal) as per manufacturer instructions. As previously mentioned, *B. ovis* infection was screened using conventional PCR with primers and conditions described elsewhere (Aktaş et al., 2005). PCRs were performed in 25 μl reactions with Supreme NZYTaq 2 × Green Master Mix (NZYTech), 1 μM primers and 5 μl of template DNA. A negative control with water and a positive *B. ovis* (Israeli strain) control were added. The PCR was carried out with a thermal cycling profile of 95°C for 2 min, and 35 cycles of 95°C for 30 s, 62°C for 45 s and 72°C for 45 s, followed by a 72°C extension for 5 min, in a T-100® Thermal Cycler (Bio-Rad, CA, USA). Resulting amplicons were checked on a 0.5X TBE, 1.2% (w/v) agarose gel.

#### Synthesis of dsRNA

Specific primers containing T7 promoter sequences (5′-TAATACGACTCACTATAGGGTACT-3′) at the 5′- end were manually designed using as template available sequences, in particular, GACK01008016 from *Rhipicephalus pulchellus*, GBBO01000019 from *Rhipicephalus microplus*, GBBR01000108 from *R. microplus*, and GACK01007634 from *R. pulchellus* and synthesized by StabVida (Lisbon, Portugal) (Supplementary Table 2). *R. bursa* cDNA was synthetized using the iScript cDNA synthesis (Bio-Rad) following the manufacturer instructions and further used as template to amplify fragments of interest by PCR. Amplifications of target DNA fragments were achieved using the iProof High Fidelity PCR kit (Bio-Rad) in a 50 μl of final volume reaction, including 200 mM of each primer. Cycling conditions were for 40 cycles: 30 s at 94°C, 30 s at specific annealing temperature and 30 s at 72°C with a final extension step of 7 min at 72°C (Supplementary Table 2). All PCR assays were performed in a T100 thermal cycler (Bio-Rad). Amplification results were analyzed on a 0.5x TBE, 1.2 % (w/v) agarose gel. Amplicons were purified using the NZYGelpure kit (NZYtech) and sent for Sanger sequencing at StabVida (Lisbon, Portugal). The obtained sequences were aligned and compared to reference sequences. After validation of the amplified sequences the MEGAscript RNAi Kit (Ambion, Austin, TX, USA) was used to synthesize dsRNA according to manufacturer's instructions. The resulting dsRNA was purified and analyzed by spectrometry and agarose gel.

#### Inoculation of dsRNA and tick infestation

*R. bursa* adult female ticks from the established colony at CEVDI/INSA were cleaned and placed ventral side up on double sticky tape, affixed to a plane wood table. Thirty female ticks per group were injected in the trochanter-coxae articulation with 69 nL of gene specific dsRNA (1 × 10^11^ to 1 × 10^12^ molecules) or unrelated dsRNA as control, using the nano-injector (Nanoject, Drummond Scientific, PA, USA). The mouse β-*2-microglobulin* dsRNA (dsβ*2M*) (GenBank: NM_009735) was used as control (Couto et al., 2017). After dsRNA injection, female ticks were held in a humidity chamber for 4 h after which they were allowed to feed on the splenectomized lamb infected with *B. ovis* together with 30 male ticks per feeding cell. Tick-feeding cells (450 × 400 mm) (cotton fabric) were glued to shaved skin using Pattex® contact glue (Henkel Nederland, Nieuwegein, Netherlands) on the day before infestation. Ticks were monitored daily and allowed to feed in the infected lamb for 8 days. After this period, attached ticks were manually removed.

#### Analysis of tick biological parameters after gene knockdown

Tick mortality was evaluated as the ratio of dead ticks to the total number of initial ticks. To analyze tick mortality, the Chi-square test (*P* > 0.05) was used with the null hypothesis that tick mortality was independent of gene knockdown. The ability to attach to the vertebrate host was also evaluated as the ratio of attached ticks and the total number of live ticks. The Chi-square test (*P* > 0.05) was also used in this analysis. Tick weight was determined in individual female ticks collected after feeding and further compared between ticks injected with test genes dsRNA and control dsRNA by Student's *t*-test with unequal variance (*P* > 0.05).

#### Gene knockdown assessment and determination of *B. ovis* infection by qPCR

To assess gene knockdown efficiency in tick SG ten ticks per group were randomly selected and tissues dissected and further used to extract total RNA and DNA and synthetize cDNA, as described previously. Quantity and quality of the RNA samples was estimated using the QIAxcel Advanced system (Qiagen™, Hilden, Germany). qPCR assays were performed under the conditions aforementioned. Gene expression was analyzed by the CFX Manager™ Software (Bio-Rad) as previously referred. Infection levels in tick SG were estimated using qPCR by evaluation of the levels of *B. ovis 18S* ribosomal DNA (*18S* rRNA) normalized against tick *16S* rDNA, as described previously for other *Babesia* spp. (Antunes et al., 2012). The primers used for detection of *B. ovis* were the same used previously for conventional PCR. The cycling conditions are described in the Supplementary Table 1. Normalized Cq-values were compared between ticks injected with dsRNA and control ticks by Student's *t*-test with unequal variance (*P* > 0.05).

#### Antigenicity prediction

Antigenicity of the selected molecules was estimated *in silico* using VaxiJen Server (Doytchinova and Flower, 2007) to allow antigen classification based on the physicochemical properties of proteins without resorting to sequence alignment. Complete sequences of the proteins were retrieved from UniProt in FASTA format and antigenicity estimated using the settings of parasite as target organism and threshold level 0.4.

## Results

### Assembly and annotation of female *R. bursa* sialomes

*R. bursa* female ticks representing the three conditions were produced and used for SG dissections, which were followed by DNA and RNA extractions. RNA qualitative and quantitative analysis are summarized in Supplementary Table 3. Infection of protozoan-exposed group (Fi) was confirmed prior to experimentation, and total RNA was used in RNA-Seq analyses. Data were collected as two sets of matched 100-bp reads and quality analysis and raw read pre-processing were performed. The *de novo* assembly statistics are presented in Table 1.

**Table 1:** Assembly statistics of the six examined *Rhipicephalus bursa* sialotranscriptomes.

**Assembly**	**NFni (1)**	**NFni (2)**	**F (1)**	**F (2)**	**Fi (1)**	**Fi (2)**
# contigs (≥0 bp)	9,832	3,433	16,931	14,051	18,801	17,032
# contigs (≥1,000 bp)	1,317	345	3,200	2,143	3,224	2,655
# contigs (≥200 bp)	9,824	3,429	16,911	14,043	18,785	17,003
Total length (≥0 bp)	5,924,670	1,825,230	12,261,455	9,122,113	13,243,635	11,318,332
Total length (≥1,000 bp)	2,478,605	589,891	6,455,940	4,306,138	6,743,456	5,408,930
Total length (≥200 bp)	5,923,273	1,824,505	12,258,149	9,120,798	13,240,893	11,313,392
Largest contig	12,501	9,307	10,194	11,098	14,540	12,991
GC (%)	51.20	55.34	57.58	51.10	50.23	51.15
# N's per 100 kbp	0.02	0.00	0.00	0.00	0.02	0.25

*NFni (1) and (2) correspond to the replicates of the pools of SG mRNA collected from unfed-uninfected females; F (1) and (2) correspond to the replicates of the pools of SG mRNA from fed-uninfected females; and Fi (1) and (2) correspond to the replicates of the pools of SG mRNA from fed-Babesia ovis infected female ticks*.

A substantial increase in the number of contigs was observed in fed-uninfected samples compared to unfed-uninfected samples. The fed-*B. ovis* infected samples exhibited the highest number of contigs (Table 1). Each transcript was annotated based on BLAST results that compared the transcript to a database of reference proteins. The complete list of results can be accessed in Supplementary Datasheets 1, 2.

**Figure 1:**
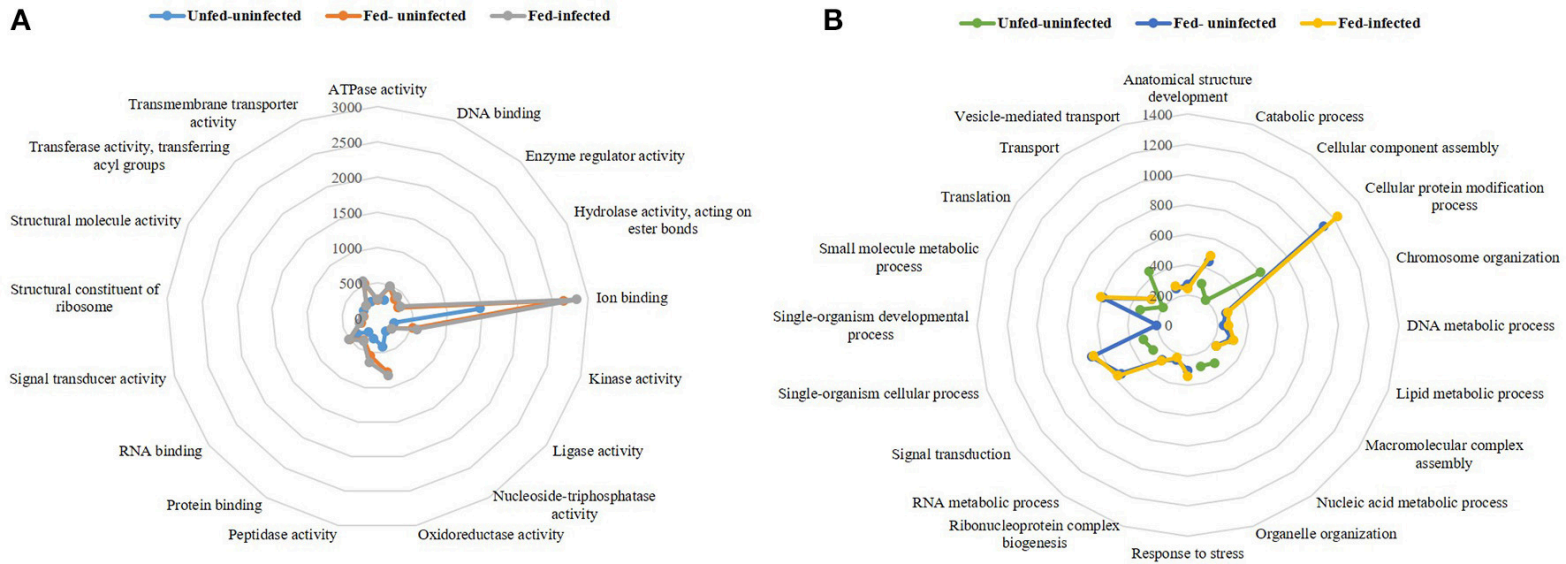
Radar plots of the three transcriptomes per represented molecular functions **(A)** and biological processes **(B)**. The lines represent a pattern of the three transcriptomes unfed-uninfected, fed-uninfected and fed-*Babesia ovis* infected, allowing a visual comparison between conditions.

The obtained transcriptomes were analyzed using the Blast2GO tool and a public Arthropoda database (nr subset) (arthropoda, taxa: 6656; from 30.01.2017). Molecular functions (Figure 1A) and biological processes (Figure 1B) of the three transcriptomes were analyzed.

The molecular functions represented in the three sialotranscriptomes included DNA, RNA, protein, and ion binding properties as well as kinase, oxidoreductase, peptidase, and transmembrane transporter activities (Figure 1A). The remaining functions represented molecular functions that were present in both fed-uninfected and fed-infected catalogs, with the exception of nucleoside-triphosphatase and structural molecule activities that were exclusive to the unfed-uninfected sialotranscriptome. Ion binding was the most represented molecular function in all three datasets (Figure 1A). Biological processes such as catabolic, cellular protein modification, single-organism cellular, small molecule metabolic processes, translation, and signal transduction were also overrepresented in all sialotranscriptomes (Figure 1B). Anatomical structure development, chromosome organization, macromolecular complex assembly, response to stress, ribonucleoprotein complex biogenesis, vesicle-mediated transport, and DNA, RNA, and lipid metabolic processes were represented in the two sialotranscriptomes associated with fed-uninfected and fed-infected conditions (Figure 1B). Single-organism development is a feeding-exclusive process, while cellular component assembly, organelle organization, transport, and nucleic acid metabolic processes were exclusive to the unfed-uninfected samples.

### Profile of SG transcriptomic dynamics in response to tick feeding and *B. ovis* infection

**Figure 2:**
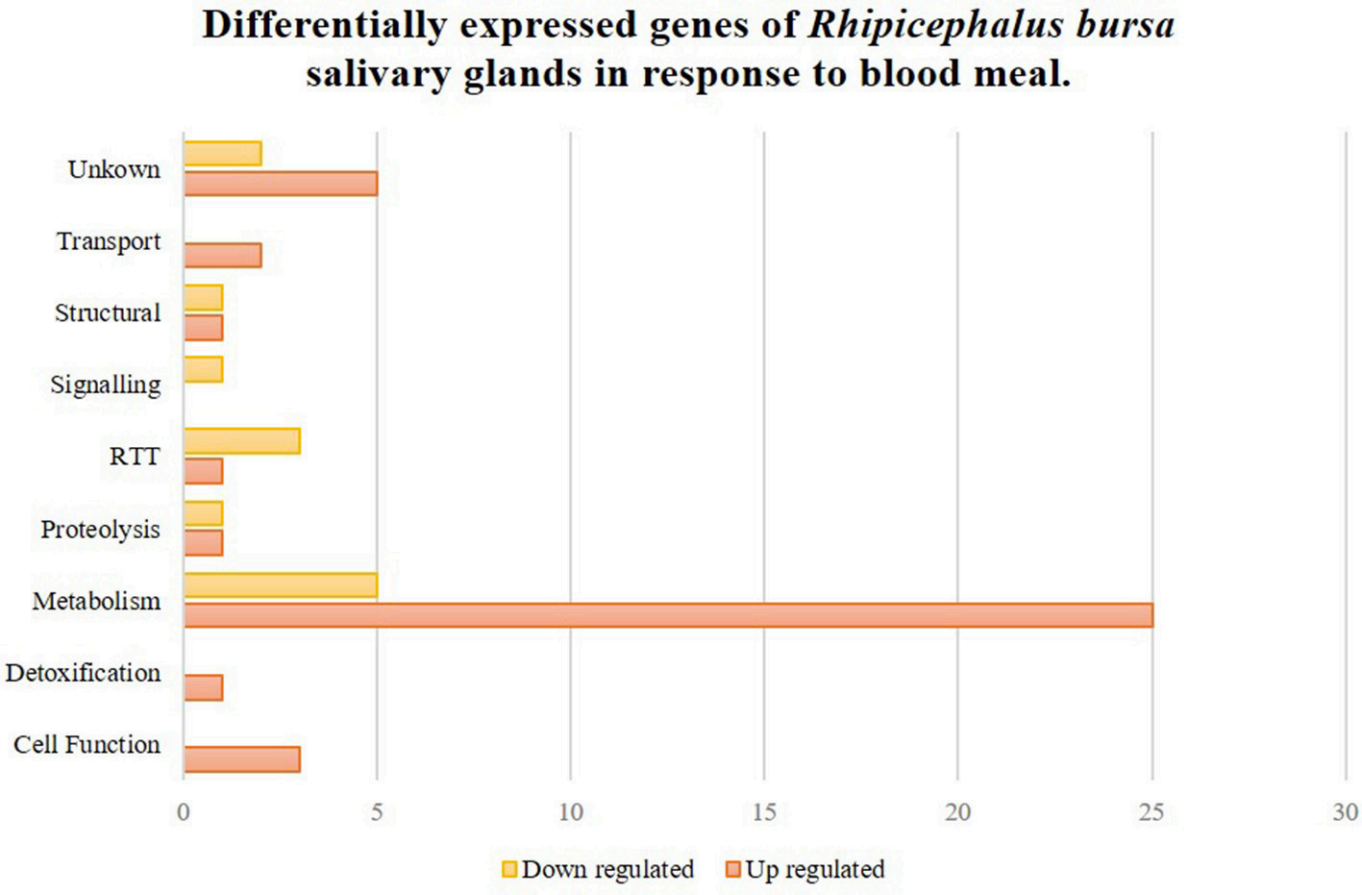
*Rhipicephalus bursa* SG transcriptional response to blood meal based on Gene Ontology functional classes assignments of encoded proteins. Yellow bars represent down regulated genes, orange bars represent up regulated genes with statistical significance (*P* < 0.05).

**Figure 3:**
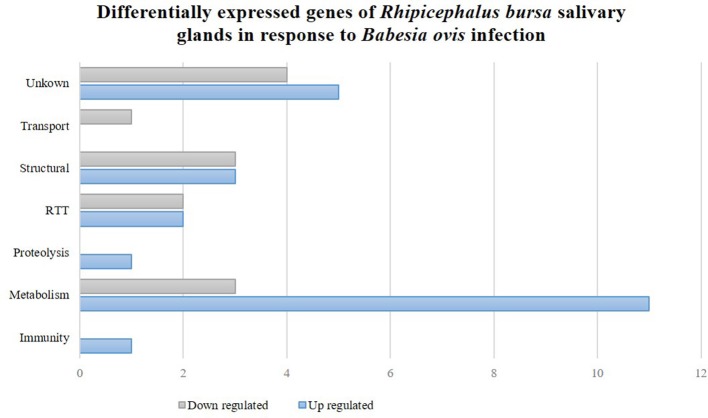
*Rhipicephalus bursa* SG transcriptional response to *Babesia ovis* infection based on Gene Ontology functional classes assignments of encoded proteins. Gray bars represent down regulated genes and blue bars represent up regulated genes with statistical significance (*P* < 0.05).

To clarify the response of *R. bursa* sialotranscriptomes to *Babesia* infection and blood feeding, an analysis that focused on the most (*P* < 0.1) up-regulated and down-regulated transcripts (Supplementary Figure 2) and as well as significantly differentially expressed (*P* < 0.05) genes (Figures 2, 3) was conducted.

In total, 7,272 and 13,819 different expressed sequence tags (ESTs) were obtained from the SG of unfed and fed ticks, respectively. From these, 5,188 were found in both conditions, 2,884 were exclusive to the unfed population, and 8,631 were only present in the SG of fed *R. bursa* females. The sialotranscriptome associated with the fed-uninfected condition was compared to the fed-infected one. The results of RNA-Seq analyses indicated that 13,819 ESTs were obtained from the sialotranscriptome of the fed sample, and 15,292 ESTs were obtained from the fed-infected sample. Of these, 9,722 ESTs were present in both samples. A total of 4,097 ESTs were exclusive to the fed-uninfected ticks, and 5,570 ESTs were only present in the SG of the fed *R. bursa* females.

Analysis of the most up-regulated and down-regulated transcripts (*P* < 0.1) (Supplementary Figure 2) indicated that 500 and 216 ESTs were differentially regulated upon feeding and infection, respectively. The diversity of molecular functions and biological processes was higher in response to blood feeding compared to infection conditions. Regarding molecular functions, hydrolase activity was the only *Babesia* infection-exclusive function, and it was completely down-regulated. The blood-feeding exclusive functions were anion, metal ion, heterocyclic, and organic cyclic compound, and protein binding activities, and these functions were only associated with up-regulated transcripts. Regarding biological processes, *B. ovis* infection resulted in the induction of biosynthetic processes, cellular protein metabolic processes, gene expression, macromolecular complex assembly, organelle organization, and symbiosis (encompassing mutualism through parasitism). However, infection was also associated with the down-regulation of catabolic processes, cellular component organization, lipid metabolic and single-organism cellular process, and transmembrane transport. *R. bursa* blood meals predominantly induced biological processes such as oxidation-reduction, organic substance biosynthetic, and cellular biosynthetic processes, and cellular amino acid metabolic process and signal transduction were down-regulated.

### SG gene differential expression in response to blood feeding

Fifty-two genes were considered significantly differentially expressed (*P* < 0.05), and these were classified based on GO for biological process and molecular functions (Figure 2). Seventy-five percent of these genes were up-regulated, and metabolism was the most up-regulated functional class in response to blood feeding. Functional classes such as transport, detoxification, and cell functions were only up-regulated, while signaling was down-regulated. Transcripts from structural, RTT (replication-transcription-translation), proteolysis, and metabolism functional classes were also differentially regulated during blood meals.

### SG gene differential expression in response to *B. ovis* infection

Thirty-six genes were considered differentially expressed (*P* < 0.05) and classified by functional classes as previously described (Figure 3). Further analyses revealed that 64 and 36% of the differentially expressed genes were up-regulated and down-regulated, respectively. Metabolism was a highly represented functional class that was associated with both up- and down-regulated genes. Structural and RTT functional classes were also affected in the *R. bursa* sialome by *Babesia* infection. Proteolysis and immunity were exclusively up-regulated, while transport was down-regulated.

### Validation of RNA-Seq results

**Figure 4:**
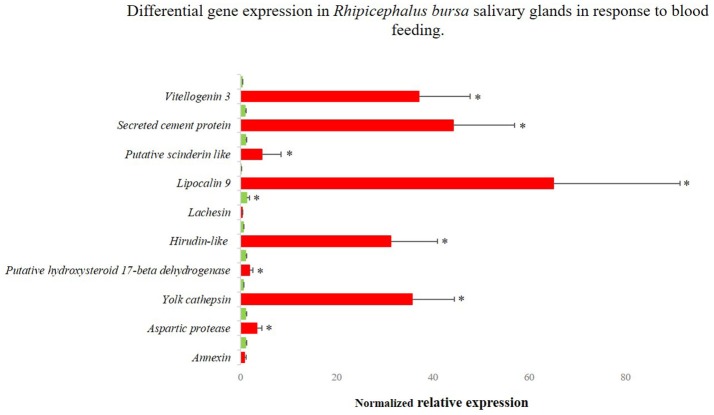
Differentially gene expression of *Rhipicephalus bursa* SG in response to blood feeding evaluated by qPCR. Red bars represent SG from fed *R. bursa* ticks and green bars represent the SG from unfed *R. bursa* ticks. ^*^P < 0.05.

**Figure 5:**
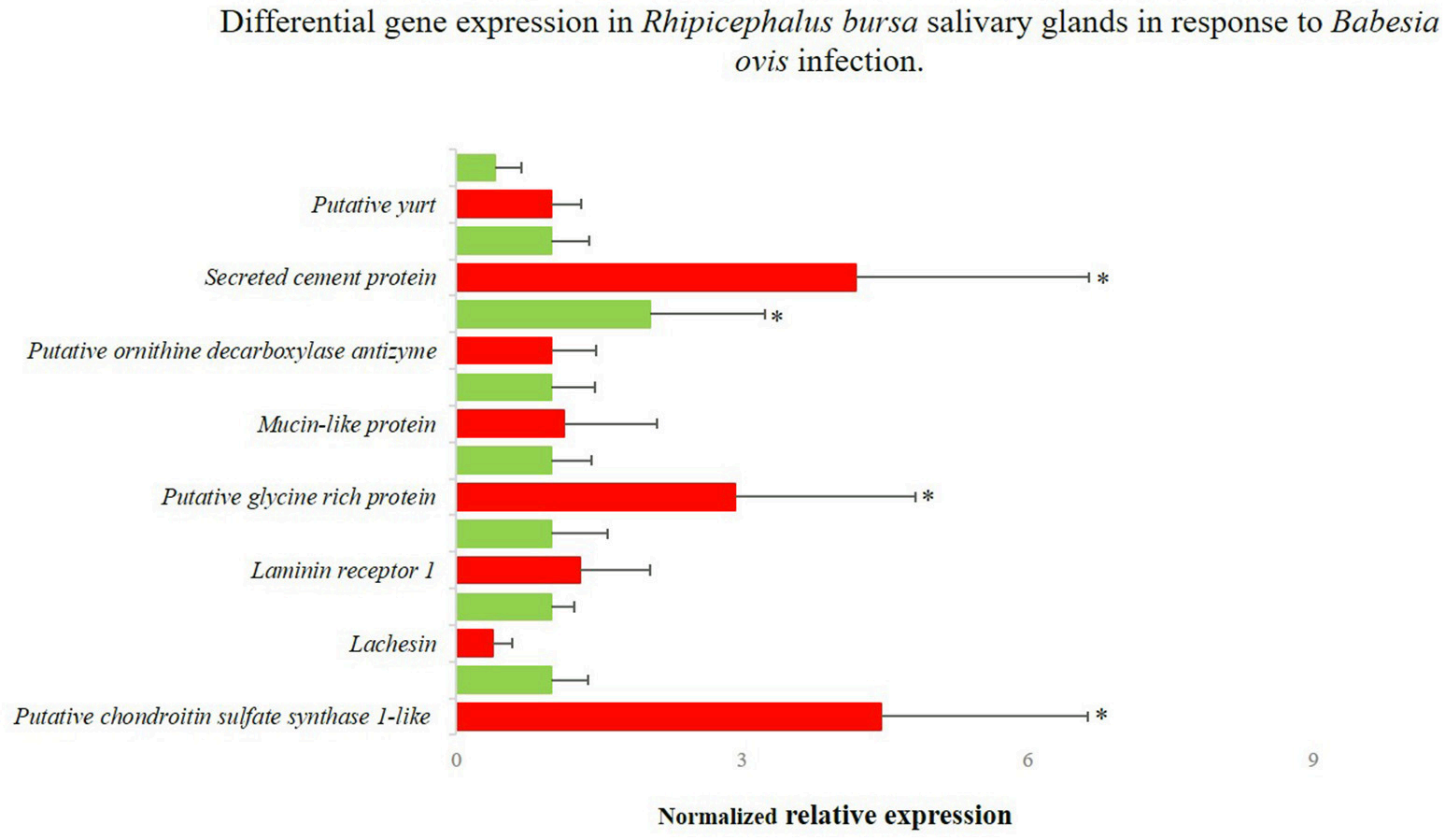
Differentially gene expression of *Rhipicephalus bursa* SG in response to *Babesia ovis* infection evaluation by qPCR. Red bars represent the *B. ovis* infected SG and green bars represent the SG from uninfected *R. bursa* ticks. ^*^P < 0.05.

Sixteen genes identified as differentially expressed in response to infection and blood feeding in RNA-Seq were selected for data validation by qPCR analysis. From the RNA-Seq catalog derived from the comparison of fed vs. unfed populations, nine transcripts that encoded the following proteins were selected: annexin (UniProt ID: A0A023FX57), aspartic protease (UniProt ID: Q2WFX6), yolk cathepsin (UniProt ID: Q56CZ1), a putative hydroxysteroid 17-beta dehydrogenase (UniProt ID L7M196), hirudin-like (UniProt ID: F0JA28), lachesin (UniProt ID: L7M018), lipocalin 9 (UniProt ID: A0A034WWJ8), a putative scinderin-like (UniProt ID: L7MCZ6), and vitellogenin-3 (UniProt ID: A0A034WWF8) (Figure 4). Regarding the RNA-Seq data obtained from the comparison of infected and uninfected SG, eight genes encoding the following proteins were selected: a putative chondroitin sulfate synthase 1-like (UniProt ID: V5H7Q8), lachesin (UniProt ID: L7M018), laminin receptor (UniProt ID: E2J6W6), a putative glycine rich protein (UniProt ID:L7M1K6), a mucin-like protein (UniProt ID: C9W1L9), a putative ornithine decarboxylase antizyme (UniProt ID: A0A023FCB3), a secreted cement protein (UniProt ID: A0A034WWS7), and a putative yurt (UniProt ID: V5HE08) (Figure 5). A moderate positive correlation between the mRNA levels by both RNA-Seq and qPCR methods was obtained (Pearson's correlation coefficient 0.5394, *P* = 0.025).

### Selection of genes for RNA interference studies

Genes for RNAi functional studies were selected based on their potential role in the condition studied and fold change of expression. The gene encoding a putative *vitellogenin*-3 (*Vg-3*) was identified herein as up-regulated in response to feeding in both RNA-Seq (fold-change 17.51, *P* = 0.025) and qPCR (fold-change 98.05, *P* < 0.001) evaluations. The GO analysis assigned the encoded protein to a lipid transporter activity function (molecular function), belonging to the lipid transport biological process. *Lachesin*, which was also selected for functional analysis, was found to be up-regulated in the RNA-Seq analysis (fold-change = 15.14, *P* = 0.045) in response to blood feeding, while it was down-regulated based on the qPCR analysis (fold-change = −3.83, *P* < 0.001). This gene was also identified in the transcriptomic response to infection (fold-change = −0.80, *P* = 0.857), so its expression during *B. ovis* infection was also verified by qPCR (fold-change = −2.6427, *P* = 0.955). Lachesin belongs to the UniRef90_A0A1E1X7K6 cluster that is related to neural cell adhesion molecules. The gene designated as *secreted cement* encodes a component that is potentially involved in cement cone formation and tick attachment, and it was up-regulated in response to infection based on both RNA-Seq (fold-change = 15.73, *P* = 0.0298) and qPCR (fold-change = 4.197, *P* = 0.007) results. The expression of this secreted cement protein was also characterized by qPCR in response to blood feeding, indicating high up-regulation (fold-change = 47.4, *P* < 0.0001) in accordance with its role in the feeding process. Lastly, an uncharacterized gene designated as *glycine rich* that encodes a putative glycine rich protein was selected from the catalog associated with infection response, and it was up-regulated based on the results of both RNA-Seq (fold-change = 14.76, *P* = 0.0382) and qPCR (fold-change = 2.931, *P* = 0.016) analyses.

### Functional analyses of differentially expressed tick genes in response to feeding and *B. ovis* infection

#### Tick attachment, weight and survival rate after RNAi

After dsRNA injection, biological parameters such as tick mortality, attachment, and weight were determined and statistically analyzed (Table 2).

**Table 2:** Evaluation of tick mortality, attachment, and weight after dsRNA injection in *Rhipicephalus bursa* ticks.

**Group**	**% of mortality rate (N)**	**% of the ticks completing feeding (N)**	***R. bursa* weight (mean ± s.d.; mg/tick)**
*vitellogenin-3*	76.67 (23)^*^	57.14(4)^b^	40 ± 19^b^
*lachesin*	70.00 (21)^*^	88.89(8)	149 ± 108
*glycine-rich*	36.37 (11)	57.90(11)^a^	73 ± 72^a^
*secreted cement*	20.00 (6)	54.20(13)^*^	52 ± 46^*^
Control	16.67 (5)	88.00(22)	136 ± 163

*Thirty female ticks per group were injected with dsRNA or unrelated dsRNA. Ticks were allowed to feed in three separated patches on a lamb experimentally infected with Babesia ovis. All attached ticks were removed after seven days of feeding, weighed, and held in a humidity chamber for four days to allow ticks to digest the blood meal. Tick mortality was evaluated as the ratio of dead ticks to the total number of ticks placed on the lamb using Chi-square test*(.

* *P < 0.05). Female tick weight after feeding was compared between dsRNA and unrelated dsRNA ticks by Student's t-test. ds, double-stranded*.

a *No statistical analysis was performed due to no gene knockdown*.

b *No statistical analysis was performed because of the insufficient number of samples*.

RNAi assays indicated that tick survival was significantly affected in dsRNA-injected ticks, in both ds*vitellogenin* (7/30; Chi-square, *P* < 0.001) and ds*lachesin* (9/30; Chi-square, *P* < 0.001) groups compared to controls (25/30), suggesting that these genes may play an important role in tick survival. The ds*vitellogenin* group was most affected with the highest mortality rate (76.67%). As represented in Table 2, the ds*cement* group was the most significantly affected by RNAi (*P* = 0.008), as 45.8% of the ticks were not able to correctly attach to the vertebrate host to complete blood meal. The ds*lachesin* injected population mimicked the control group's ability to attach to the host and feed. The average body weight was also measured, and it was significantly higher in the control group (133 ± 119 mg) than the *Vg-3*-silenced group (40 ± 19 mg); however, no statistical study was conducted because of the low number of ticks (N = 4). *Lachesin* knockdown did not affect tick weight (149 ± 108 mg) (*P* > 0.05). The knockdown of the gene encoding the cement protein significantly reduced female weight (52 ± 46 mg, *P* = 0.021) and only 13 ticks were able to attach to the host.

#### Gene silencing efficiency and *Babesia* infection evaluation

Under the studied conditions, dsRNA-mediated gene knockdown efficiency and its effect on *B. ovis* infection was assessed (Table 3).

**Table 3:** Efficiency of gene knockdown by RNA interference and its influence on *B. ovis* infection levels in *Rhipicephalus bursa* ticks SG.

**Group**	**Gene silencing (% Ave ± *S.D*.)**	***B. ovis* infection levels (Ave ± *S.D*.)**	**Infection rate (Ave ± *S.D*.) (N)**
*vitellogenin-3*	92 ± 2^*^	4.67e^−04^ ± 3.05e^−04^	314 ± 255.17(4)^a^
*lachesin*	51 ± 9^*^	4.48e^−07^ ± 1.20e^−07^	0.30 ± 0.09(8)^*^
*glycine-rich*	ND	–	–
*secreted cement*	65± 11^**^	2.97e^−06^ ± 2.68e^−06^	1.99 ± 2.18
Control	–	1.49e^−06^ ± 1.09e^−06^	–

*Thirty female ticks per group were injected with dsRNA or unrelated dsRNA. Ticks were allowed to feed in six separated patches on a lamb experimentally infected with Babesia ovis. All attached ticks (n = 4–22) were removed after seven days of feeding and held in a humidity chamber for four days to allow ticks to digest the blood meal. Gene knockdown was analyzed by qPCR by comparing mRNA levels between specific dsRNA-injected and control ticks using the CFX Manager™ Software by means of the Pfaff method*,

* P < 0.05. The B. ovis infection levels were determined by qPCR of the pathogen 18S rRNA gene and normalized against tick 16S rRNA using the ddCq method ($2_{\text{target}}^{-\text{Cq}}$${}_{\text{control}}^{-\text{Cq}}$). Infection rate was calculated by the ratio of silenced per control groups. The mRNA levels and B. ovis infection in ticks were compared between specific dsRNA injected and control ticks by a Student's t-test (^*^P < 0.05;

** *P < 0.01). ds, double-stranded; ND, not demonstrated*.

a *No statistical analysis was made due to the insufficient number of samples*.

The injection of dsRNA molecules in *R. bursa* ticks led to a significant reduction of *vitellogenin, lachesin*, and *secreted cement* mRNA levels in SG by 92% (*P* = 0.040), 51% (*P* = 0.047), and 65% (*P* = 0.018), respectively. Regarding the levels of infection acquired after feeding on an experimentally *B. ovis*-infected lamb, the results indicated that the knockdown of *lachesin* significantly reduced *B. ovis* infection levels by 70% (*P* = 0.00251) in *R. bursa* SG (Table 3). The remaining groups exhibited increased infection levels.

Antigenicity of vitellogenin-3, lachesin, and secreted cement proteins were predicted by VaxiJen tool selecting parasite as the target organism. The three proteins showed to be probable antigens.

## Discussion

Babesiosis is one of the most important diseases transmitted by ticks that affect a wide range of vertebrates, considered an emerging zoonose (Hunfeld et al., 2008; Ord and Lobo, 2015; Antunes et al., 2017). *B. ovis* is a potentially lethal pathogen that is normally found in small ruminants, and it is primarily transmitted by *R. bursa*, a tick species that is widely distributed in the Mediterranean region (Walker et al., 2000; Ferrolho et al., 2016a). Despite the importance of the *R. bursa*-*B. ovis*-vertebrate host interactome, no studies have examined these molecular relationships. Although it is recognized that transcripts and protein levels in ticks do not always correlate because of post-transcriptional and post-translational modifications (Ayllón et al., 2015; Villar et al., 2015), transcriptomic analysis is essential for a proper understanding of the molecular constituents of cells and tissues and the interactions and relationship between parasites and disease development (Li and Biggin, 2015; Rokyta et al., 2015). The integration of different omics analyses have allowed the detailed characterization of tick-pathogen molecular pathways (Ayllón et al., 2015; Cabezas-Cruz et al., 2017a,b). Herein, to elucidate the cellular mechanisms behind blood feeding and *Babesia* infection, three sialotranscriptomes of *R. bursa* females were analyzed and SG genes were selected for further characterization with RNAi to assess their potential as tick protective antigens.

### Overall characteristics of the *R. bursa* sialome in response to blood feeding and *Babesia* infection

A strong transcriptional response was induced after tick feeding and during *B. ovis* infection, since a higher and more diverse number of transcripts were detected in the fed-uninfected sample, and even more diverse transcripts were detected in the fed-infected samples (Table 1 and Figure 1A) in comparison with the unfed-uninfected SG samples. This type of response was previously described in other systems (Heekin et al., 2013; Tirloni et al., 2014; Ayllón et al., 2015; Villar et al., 2015; de Castro et al., 2016, 2017; Kim et al., 2016; Perner et al., 2016; Valdés et al., 2016; Schroeder et al., 2017), thus indicating that different tick biological processes or statuses stimulate different gene expression regulatory strategies.

Functional annotation indicated that in all transcriptomes, ion binding molecular function was the most represented category, and its representation nearly doubled in response to feeding (Figure 1A). Being obligatory hematophagous ectoparasites, ticks must deal with the iron and heme resulting from blood catabolism. Ticks are known to express iron and heme binding proteins that sequester excess iron or heme, preventing cell damage for physiologically normal cells (Galay et al., 2015; Kim et al., 2016).

Structural molecule activity is the only class more represented in the unfed-uninfected SG transcriptome, while other molecular function categories such as structural constituent of ribosome or enzyme regulator activity are exclusive to the fed-uninfected and fed-infected populations that exhibit high cellular activity (Villar et al., 2014).

The most represented biological process in all sialomes was the cellular protein modification. The transcript abundance of transcripts belonging to this biological process doubled in the fed-uninfected and fed-infected SG samples in comparison to the unfed-uninfected ones. The anatomical structure development process was only represented in the fed populations, and this possibly reflected SG enlargement during feeding as the majority of acinar cells undergo marked hypertrophy in Ixodid females (Šimo et al., 2017). Furthermore, some pathogens induce cytoskeletal rearrangement by affecting the regulation of specific mRNAs (Ayllón et al., 2013, 2015; Ireton, 2013; Cotté et al., 2014; de la Fuente et al., 2017). As expected, metabolism-related processes were markedly represented in the transcriptomes of fed samples. The response to stress was only identified in the fed-uninfected and fed-infected SG samples, and this was in accordance with previous studies that indicated high regulation of such pathways in ticks and cells infected with *Anaplasma* spp. (Villar et al., 2010, 2014) and during feeding (oxidative stress response) (Kim et al., 2016). The unfed sialotranscriptome profile revealed the maintenance of basal cellular metabolism (Figure 1B). Lipid metabolic processes were exclusively represented in the fed-uninfected and fed-infected samples, thus correlating with higher cellular energy requirements and saliva production (Denardi et al., 2011). Being a cellular energy source, lipids in tick SG are implied in cement cone formation, thus explaining the high representation of such metabolic activity (Denardi et al., 2011). A comparable result was obtained in *Ixodes ricinus* and *Rhipicephalus appendiculatus* SG after feeding (Kotsyfakis et al., 2015; de Castro et al., 2016). Salivary lipid interacting proteins were up-regulated in *I. ricinus* infected with *Borrelia burgdorferi* (Cotté et al., 2014) suggesting that certain pathogens can manipulate vector lipid metabolism to facilitate infection and multiplication (Perera et al., 2012; Grabowski et al., 2017).

### Specific *R. bursa* sialome response to blood feeding

Few studies have focused on the sialotranscriptomic response to tick feeding (McNally et al., 2012; Kotsyfakis et al., 2015; Yu et al., 2015; de Castro et al., 2016, 2017; Maruyama et al., 2017), but all demonstrated that transcription was highly affected in SG. Kotsyfakis et al. (2015) showed that fed *I. ricinus*, SG exhibit 10 times more overexpression compared to the midgut. Herein, genes that were highly differentially expressed in response to blood meals indicated up-regulation at rates of 75.0% (*P* < 0.05) to 83.8% (*P* < 0.1). GO analyses revealed that expression of secreted proteins was induced during tick feeding, including 14 lipocalins, four metalloproteases, two glycine rich proteins, and three microplusins (Supplementary Datasheet 1, Supplementary Figure 2). Such transcriptional regulation differs throughout tick feeding, thus reflecting the necessity of the tick to first attach to the host, evade and modulate host immune defenses, and maintain this status during the prolonged feeding period (McNally et al., 2012; Kotsyfakis et al., 2015; Chmelar et al., 2016; de Castro et al., 2017). Furthermore, fatty-acid related transcripts were highly represented in the up-regulated SG genes, suggesting a significant investment in carbohydrate metabolism. After tick attachment, SG differentiate and convert from an inactive to a metabolically active status with intense biosynthesis of molecules and ion transport, which increase cell energy requirements (McNally et al., 2012). The most up-regulated transcripts identified herein using RNA-Seq analyses encoded a fatty acid synthase (fold-change = 17.67), followed by vitellogenin-3 (fold-change = 17.51) and a glycine-rich cell wall structural protein (fold-change = 17.51). Two uncharacterized proteins (fold-changes = −17.66 and −16.63) and two glycine rich proteins (fold-changes = −16.63 and −15.71) encoded transcripts were highly down-regulated (Supplementary Datasheet 1). These results suggested that in the late stage of feeding, female ticks switch the regulation of specific proteins related to the production of cement cone, thus driving drop-off in accordance with previous reports (McNally et al., 2012; Kotsyfakis et al., 2015; de Castro et al., 2017).

### Specific *R. bursa* sialome response to *B. ovis* infection

The sialotranscriptomes of fed-infected and fed-uninfected female *R. bursa* were compared to characterize SG transcriptional regulation in response to pathogen infection. As all of the SG samples belonged to fed ticks, the effect of the feeding process can be annulled. Some studies aimed to understand the effects of pathogens on tick SG at transcriptomic, proteomic, and metabolomic levels (Nene et al., 2004; Zivkovic et al., 2010; Mercado-Curiel et al., 2011; McNally et al., 2012; Cotté et al., 2014; Ayllón et al., 2015; Villar et al., 2015; Valdés et al., 2016). Because of their medical importance, many of these studies were dedicated to *Anaplasma* spp./*Borrelia* spp.*-Ixodes* spp. interactions This is the first study that specifically focused on the *Rhipicephalus* SG transcriptomic response to *Babesia* infection. Pathogens highly adapted to the vector such as *Anaplasma*-*R. microplus* do not induce great effects on SG, while pathogens that pose a higher threat to vector fitness would lead to a greater gene modulation (Cen-Aguilar et al., 1998; Zivkovic et al., 2010; Mercado-Curiel et al., 2011; Chmelar et al., 2016; de la Fuente et al., 2016; Šimo et al., 2017). In *Babesia* infections, tick development tends to be impaired, but adaptive parasite tolerance has been described in *R. microplus* (Cen-Aguilar et al., 1998; Antunes et al., 2017). Furthermore, a small number of genes were considered differentially expressed (36 genes at *P* < 0.05 and 260 genes at *P* < 0.1), suggesting the long co-evolution of *R. bursa* and *B. ovis*. In both analyses an up-regulation of 63–64% of the genes occurred. Our results showed that during *Babesia* invasion, cellular metabolism tended to increase, whereas biosynthesis and protein processing were the most represented categories (Supplementary Datasheet 2, Supplementary Figure 2). This metabolism induction was previously demonstrated in other vector-pathogen systems (Mercado-Curiel et al., 2011; Heekin et al., 2012; Ayllón et al., 2015; Villar et al., 2015). The most up-regulated genes found were related to glycine-rich proteins (GRPs), including uncharacterized protein (fold-change = 17.53), glycine rich proteins (fold-change = 16.45 and 15.65), and secreted cement protein (fold-change = 15.73). Glycine rich proteins have been identified as upregulated in response to infection and cement proteins (Nene et al., 2004; Zivkovic et al., 2010). With rare exceptions, the role of such proteins during pathogen infection/dissemination have not been investigated (Trimnell et al., 2002). Lipocalins and defensins were identified as up-regulated in our dataset, showing an investment of the tick in the immune response, as expected. To validate the RNA-Seq results, qPCR was employed targeting putatively down-regulated *ornithine decarboxylase antizyme, lachesin*, and *chondroitin sulfate synthase* genes and putative upregulated *laminin receptor, yurt, glycine rich, secreted cement*, and *mucin*. *Chondroitin sulfate synthase* and *lachesin* expression trends were not confirmed, indicating up-regulation in infected SG. Chondroitin's are known to be involved in *Plasmodium* spp. adhesion to cells (Dinglasan et al., 2007; Couto et al., 2017), so the up-regulation of related molecules in infected tick SG suggests that *Babesia* spp. (considered a *Plasmodium*-like parasite) may use similar strategies to invade cells.

### Functional studies for the identification of tick protective antigens

#### Vitellogenin-3

**Figure 6:**
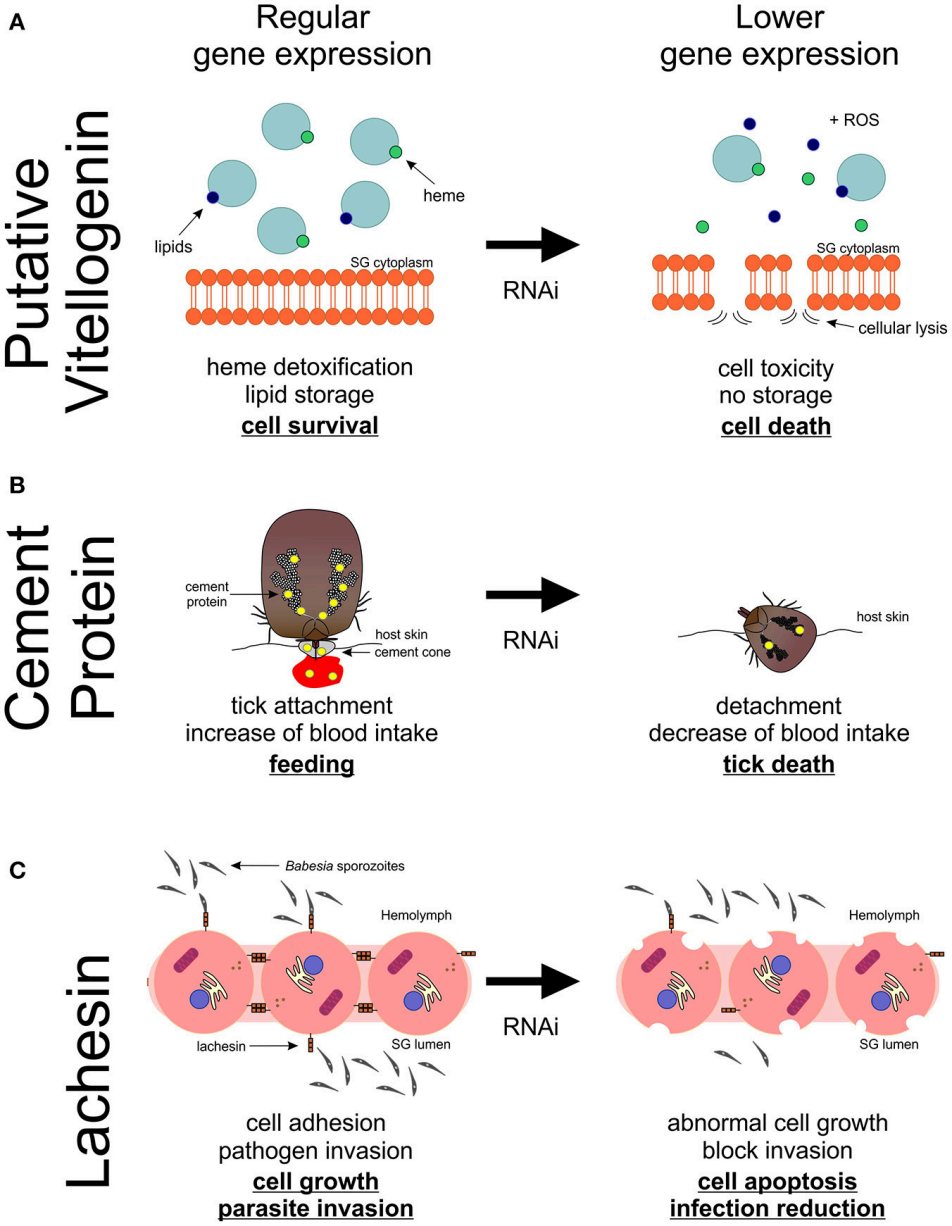
Proposed model of putative vitellogenin-3, cement protein and lachesin functions and its impact on *Rhipicephalus bursa* SG during feeding and *Babesia ovis* infection. **(A)** Vitellogenin-3 described function relates to heme detoxification and lipid storage contributing for cell survival. A decrease of the expression of putative *vitellogenin-3* leads to deficient heme seizure, increasing the formation of reactive oxygen species (ROS) as well as cellular toxicity. Lipid storage is also compromised leading to an unbalance in the production of energy. **(B)** Putative cement protein is a component of the cement cone, which facilitates the tick attachment and feed on the host. An impact in the production of cement proteins leads to an incapacity of ticks to correctly attach and subsequently feed on the host, resulting in tick death and reduced blood ingestion. **(C)** Lachesin is a cell surface protein that as a potential role in cell adhesion, maintaining apical-basal polarity, vesicle trafficking, cell growth and survival, as well as parasite invasion. A negative manipulation of the expression of *lachesin* results in an abnormal cell growth and ultimately cell apoptosis, and also a decrease of *Babesia* spp. infection.

Multiple vitellogenins (Vgs) have been described in ticks (Thompson et al., 2007; Boldbaatar et al., 2010; Khalil et al., 2011; Taheri et al., 2014; Rodriguez et al., 2016), and they are involved in detoxification and oxidative molecular processes (Galay et al., 2015). In the sialotranscriptome obtained in response to blood feeding, the translation of one of the assembled transcripts showed high similarity to *R. microplus* putative Vg-3 protein (UniProt ID: A0A034WWF8). An up-regulation of the expression of the correspondent gene in the SG of fed *R. bursa* was demonstrated by both RNA-Seq and qPCR (RNA-Seq: fold-change = 17.509, *P* = 0.025; and qPCR fold-change = 98.05, *P* < 0.001), and the results were in accordance with those of previous studies (Horigane et al., 2010; Yang et al., 2015). Vgs are thought to be absent from SG, whereas heme transport and storage are thought to be dependent of the hemelipoglyco-carrier protein (CP) (Donohue et al., 2009). In ticks, both Vg proteins and CP bind heme (Logullo et al., 2002), which is a functional component of many hemoproteins, but it is cytotoxic in larger amounts (Ferrolho et al., 2016b; Hajdusek et al., 2016). The similarities between CPs and Vgs in ticks, as well as their common evolutionary origin, greatly complicate their differentiation and function assignments (Gudderra et al., 2002; Donohue et al., 2009; Boldbaatar et al., 2010). The present study showed that *R. bursa* possesses a gene very similar to *Vgs* in SG, and it shares several molecular features with CPs. Further studies are necessary to clarify Vgs classification in ticks as well as the function and localization of *Vg-3* in *R. bursa* species as these *Vgs* are expressed in a tissue-specific manner in ticks (Rodriguez et al., 2016). *Vg-3* knockdown experiments resulted in increased tick mortality. No statistical analyses were performed regarding feeding behaviors, body weight and *Babesia* infection, because of the low number of samples; however, decreased blood-uptake and increased *Babesia* infection was observed. Based on the principal functions associated to this type of molecule, we can suggest that a decrease in the expression of putative *Vg-3* reduces heme and lipid binding and storage (Figure 6A). A deficient heme seizure may increase cellular toxicity, thus contributing for the formation of reactive oxygen species (ROS). Also, the role of Vgs on lipid transport is compromised, and this may unbalance normal energy production. Vgs have been consistently discovered has highly immunogenic molecules in *Rhipicephalus* ticks (Boldbaatar et al., 2008, 2010; Smith and Kaufman, 2013; Taheri et al., 2014; Rodriguez et al., 2016), and the results of the present study stimulates future research.

#### Putative secreted cement protein and glycine-rich protein

The genes encoding putative cement protein and GRP were found to be significantly up-regulated in response to *B. ovis* infection in *R. bursa* SG, in accordance with a previous study (Nene et al., 2004). The cement cone is composed of several molecules that are embedded in a proteinaceous matrix, presenting several GRPs (Bishop et al., 2002; Trimnell et al., 2005; Maruyama et al., 2010). Different species of ticks rely on different types and amounts of GRPs in order to attach and feed on their hosts. Briefly, ticks with short mouthparts need higher amounts of GRPs than those with long mouthparts. Moreover, one-host ticks present a greater variety of these proteins than ticks that feed on several hosts (Maruyama et al., 2010). A successfully knockdown was observed in *cement*-silenced ticks, but no silencing was demonstrated in *glycine-rich* dsRNA-injected ticks, suggesting that a higher concentration may be needed to reduce the expression of this gene. The *cement*-silenced ticks significantly affected tick attachment, feeding, and body weight (Figure 6B). The ds*glycine-rich* RNA inoculated group exhibited a slight decrease in these two parameters, reflecting its potential in tick feeding capacity and attachment to the host. Curiously, in both dsRNA-injected groups, an increase of *Babesia* levels was detected. Previous studies concerning cement cone proteins showed that immunization with these proteins significantly affected tick attachment to the host (Trimnell et al., 2005) and it reduced pathogen transmission (Labuda et al., 2006). Therefore, these two proteins are attractive targets for vaccine development.

#### Lachesin

Lachesin is a cell surface protein of the immunoglobulin superfamily (Karlstrom et al., 1993; Llimargas et al., 2004) that regulates organ size by influencing cell length and cell detachments, suggesting a role in cell adhesion and connection (Llimargas et al., 2004). In ticks, the gene encoding lachesin was first identified in the genome of *Ixodes scapularis* (Gulia-Nuss et al., 2016) and more recently in the sialotranscriptome of *Amblyomma cajennense* (Garcia et al., 2014), *R. pulchellus* (Tan et al., 2015), and *R. appendiculatus* (de Castro et al., 2016). However, no studies that focus on this molecule in ticks have been performed. In the present study, an assembled transcript translated to a protein highly similar to lachesin (UniProt ID: L7M018). A highly dynamic expression profile of *lachesin* in response to infection and feeding was found in the present study, and this observation aligned to its presumed role on cell-adhesion led to its selection for RNAi studies. Tick inoculation with ds*lachesin* resulted in 51% gene knockdown that led to a significantly high tick mortality. Lachesin accumulates in specific invertebrate cell junctions, and it is responsible for establishing and/or maintaining cell polarity, cell adhesion, and cell-cell interactions (Tepass et al., 2001). Apical-basal polarity is subjected to tight regulation, as it is crucial during tissue formation, including vesicle trafficking machinery, morphogenesis, and modulation of epithelial cell growth and survival (Bonazzi and Cossart, 2011). Moreover, adhesive contacts between cells and the extracellular matrix appear as important landmarks for polarity. Therefore, manipulating the expression of genes involved in this processes can induce abnormal cell growth and cell apoptosis (Tepass, 2012). In addition, the *lachesin* knockdown resulted in lower pathogen infection in the SG. No statistical effect was demonstrated in the other biological parameters studied. Despite the tight organization of the epithelium barrier and its interactions with cellular factors that are crucial to cell-pathogen defense, a large number of pathogens have developed strategies to target host proteins involved in cell adhesion, to colonize epithelia, invade host cells, or even disrupt host barriers to facilitate access to other tissues (Bonazzi and Cossart, 2011). Thus, our results suggest that lachesin plays an important role in tick survival and also that *B. ovis* may require this molecule for tissue invasion (Figure 6C). This molecule appears to be good candidate for future vaccination assays, as it demonstrates a dual-effect targeting both tick and pathogen.

## Conclusions

Tick and tick-borne diseases constitute a growing burden for human and animal health, stressing the urgency in the development of new effective tools to control this global threat. Due to the important role of tick SG in tick biology and pathogen transmission, the main objective of the present study was the identification and functional characterization of *R. bursa* SG genes involved in tick feeding and *B. ovis* infection. Quantitative transcriptome analysis showed *lachesin* and putative *vitellogenin-3* has highly upregulated in response to blood meal and the genes encoding for a putative secreted cement and GRPs highly upregulated in response to *B. ovis* infection. RNAi studies suggest that *lachesin* and putative *vitellogenin-3* affect tick survival while the putative cement protein has an impact in tick attachment to the host and tick weight after feeding. Moreover, *B. ovis* infection levels in tick SG were reduced, subsequently to *lachesin* knockdown. Overall the results of the present study endorse the inclusion of these proteins in vaccination trials.

## Author contributions

SA, JdlF, MMS-S, and AD designed the study; ASS and MMS-S were responsible for tick rearing and tick inoculation methodology. SA established and maintained *B. ovis* cultures. SA, JC, and JF performed transcriptomic analyses; SA and FR performed qPCR assays; SA, FR, JF, and JN performed the RNA interference studies; SA, JC, JdlF, and AD performed data analysis and wrote the manuscript. All authors edited and approved the final manuscript.

### Conflict of interest statement

The authors declare that the research was conducted in the absence of any commercial or financial relationships that could be construed as a potential conflict of interest.
