# Supplementary figures and images for: Rhipicephalus bursa Sialotranscriptomic Response to Blood Feeding and Babesia ovis Infection: Identification of Candidate Protective Antigens
Source: Front Cell Infect Microbiol. 2018 May 4;8:116. doi: 10.3389/fcimb.2018.00116 (PMC5945973; doi:10.3389/fcimb.2018.00116)

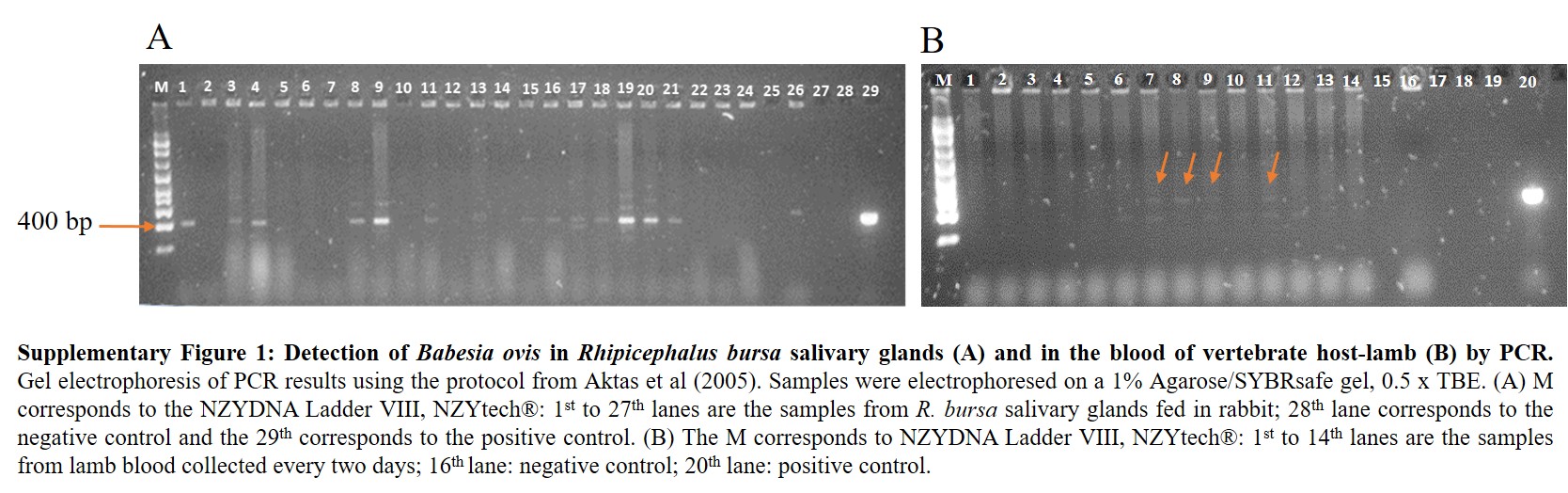

Supplement: Supplementary file 3 [file Image_1.jpg]

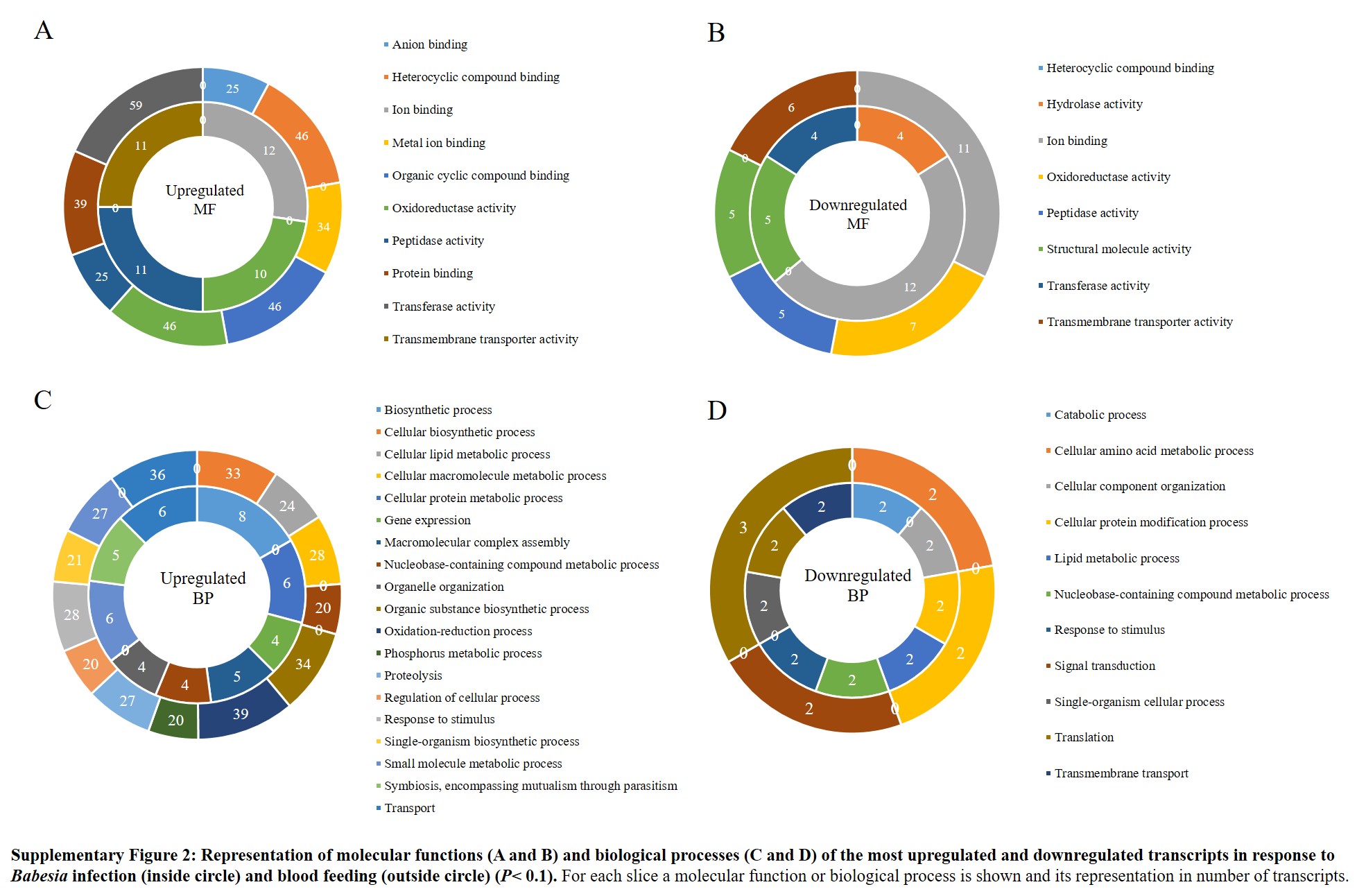

Supplement: Supplementary file 4 [file Image_2.jpg]
